# Population-Based Placental Weight Ratio Distributions

**DOI:** 10.1155/2014/291846

**Published:** 2014-05-06

**Authors:** Erin M. Macdonald, John J. Koval, Renato Natale, Timothy Regnault, M. Karen Campbell

**Affiliations:** ^1^Department of Epidemiology and Biostatistics, The University of Western Ontario, London, ON, Canada N6A 5C1; ^2^Department of Obstetrics and Gynecology, The University of Western Ontario, London, ON, Canada N6A 5C1; ^3^Children Health Research Institute, London, ON, Canada N6C 2V5; ^4^Department of Paediatrics, The University of Western Ontario, London, ON, Canada N6A 5C1; ^5^Department of Physiology and Pharmacology, The University of Western Ontario, London, ON, Canada N6A 5C1

## Abstract

The placental weight ratio (PWR) is a health indicator that reflects the balance between fetal and placental growth. The PWR is defined as the placental weight divided by the birth weight, and it changes across gestation. Its ranges are not well established. We aimed to establish PWR distributions by gestational age and to investigate whether the PWR distributions vary by fetal growth adequacy, small, average, and large for gestational age (SGA, AGA, and LGA). The data came from a hospital based retrospective cohort, using all births at two London, Ontario hospitals in the past 10 years. All women who delivered a live singleton infant between 22 and 42 weeks of gestation were included (*n* = 41441). Nonparametric quantile regression was used to fit the curves. The results demonstrate decreasing PWR and dispersion, with increasing gestational age. A higher proportion of SGA infants have extreme PWRs than AGA and LGA, especially at lower gestational ages. On average, SGA infants had higher PWRs than AGA and LGA infants. The overall curves offer population standards for use in research studies. The curves stratified by fetal growth adequacy are the first of their kind, and they demonstrate that PWR differs for SGA and LGA infants.

## 1. Background

Placental weight is a measure which reflects many aspects of placental growth including the laterally expanding growth of the chorionic disc, increased placental thickness with arborization of the chorionic villi, and increased surface area for vascular nutrient exchange. Thus, the growth of the chorionic disc, beginning early in pregnancy, is the main determinant of its transfer capacity, which supports the fetus in achieving its genetic growth potential [1].

Fetal growth depends on placental growth. Fetal growth restriction (FGR) is the failure of a fetus to reach his/her biological growth potential and small for gestational age (SGA) is widely used as a statistical indicator of FGR. SGA is defined as birth weight < 10th percentile for gestational age and sex based on a population standard [2]. Placental weight is generally lowered in SGA infants than in average for gestational age (AGA) and generally larger in large for gestational age infants (LGA) [3–5]. The placental weight ratio (PWR) reflects whether the relative growth of the placenta and fetus is proportionate and is a common measure of the balance between placental and fetal growth. The PWR is defined as the placental weight divided by the birth weight and decreases across gestation as the placental growth slows and fetal growth accelerates [6]. Placental hypertrophy and reduced fetal growth have been postulated to be an adaptation to maintain placental function in pregnant women with complications such as malnutrition [7]. If this is true, a pregnancy with impaired fetal growth, resulting in a SGA infant, should have an increased PWR compared to those infants who are AGA or LGA [1, 8].

Placental weight and the PWR have been found to vary with maternal conditions and, to be predictive of obstetrical outcomes, perinatal morbidity and mortality, childhood growth and development, and fetal origins of adult onset disease [9–14]. Previous studies that have looked at the relationships between PWRs and outcomes have not used a population standard to objectively identify abnormal PWRs [15–17]. It is important to have population percentile standards in order to objectively differentiate a normal from abnormal PWR. Accordingly, the first objective of this study was to develop standard curves for the PWR across gestational ages in a population-based birth cohort. Since literature evidence suggests that placental weights differ between SGA, AGA, and LGA infants, a second objective was to examine this in order to refine the potential applications of the PWR trajectories. Having normative PWR standards by gestational age and an understanding of how these differ for under grown and overgrown fetuses will provide a useful standard for further research.

## 2. Methods

**Figure 1:**
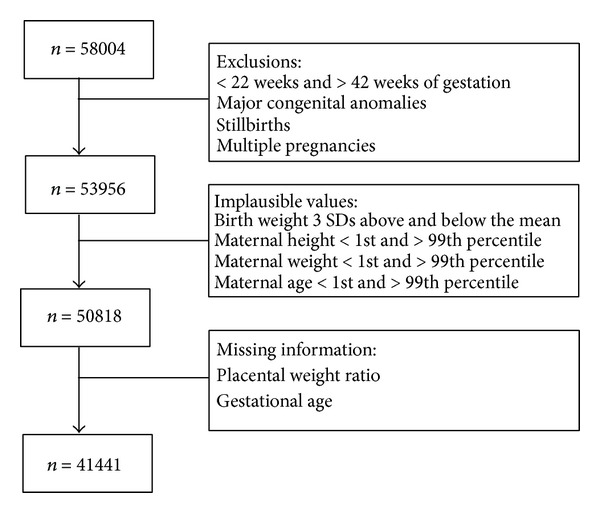
Flow chart illustrating the process by which the study population was obtained.

The study included all in-hospital singleton births in the city of London, Ontario. Research ethics board approval from Western University was obtained. Data arose from a perinatal database including all births occurring at St. Joseph's Health Care and Victoria Hospital in London, Ontario, between April 1, 2001 and March 31, 2011. A dedicated research assistant collected the information in the database from the medical charts, delivery records, and neonatal records. Placentas and infants were weighed by nursing assistants with an electronic weight scale. Placentas were weighed with the membranes and umbilical cord, including the segment of cord used for cord blood sampling. No attempt was made to remove placental blood before weighing. Anomalies (*n* = 881), stillbirths (*n* = 422), and multiple gestations (*n* = 2876) were excluded from the analyses. Placental weight was not available for 13,084 records, largely because one hospital did not collect placental weight for the entire study period. However, examination of the placental weights across gestational ages between the two hospitals revealed no significant differences. Therefore, total sample size consisted of 21,255 males and 20,186 females (total *n* = 41,441), as detailed in sample flow chart in Figure 1. Of these, 33,582 were residents of London-Middlesex while 7,859 were regional referrals. Only births between 22 and 42 weeks of gestation were included. Gestational age was truncated to the number of completed weeks based on the recommendations from World Health Organization and International Classification for Disease and was based on ultrasound or last menstrual period. Birth weight was categorized into SGA, AGA, and LGA based on Kramer et al. standards [18].

Descriptive analyses were performed on all study variables. Implausible values and potential errors were excluded including birth weights above or below the mean by three SDs, placental weights that were ≤100 g or ≥2500 g, maternal age, maternal height, prepregnancy weight below the 1st and above 99th percentiles, and any unknown or ambiguous genders.

Placental and birth weight distribution curves, and PWR curves, by gestational age were produced stratified by sex. The primary analysis estimated PWR curves for the total population, including referrals from outside the London-Middlesex. A sensitivity analysis was performed excluding regional referrals from outside London-Middlesex. This sample, hereinafter referred to as the “city-wide” sample, would be expected to produce estimates with high internal validity because they represent a “whole population” perspective. The citywide PWR distributions were compared to the total population PWR distributions inclusive of referrals in order to assess their similarity. Due to its larger size, particularly at earlier gestational ages, the sample including regional referrals was used to create PWR distribution curves separately for SGA, AGA, and LGA infants.

We created growth charts at the 3rd, 5th, 10th, 25th, 50th, 75th, 90th, 95th, and 97th percentiles using quantile regression with quadratic terms on gestational age for the PWR. Quantile regression was also used for the placental and birth weight distributions, but quadratic splines at 22, 32, and 42 weeks of gestation were used as opposed to a quadratic term for gestational age, as it allowed for a better fit of the model. Quantile regression does not impose any parametric assumptions on the response distributions, which makes it appropriate for the anthropometric measures [19].

## 3. Results

### 3.1. Placental Weight, Birth Weight, and PWR Distributions

**Figure 2:**
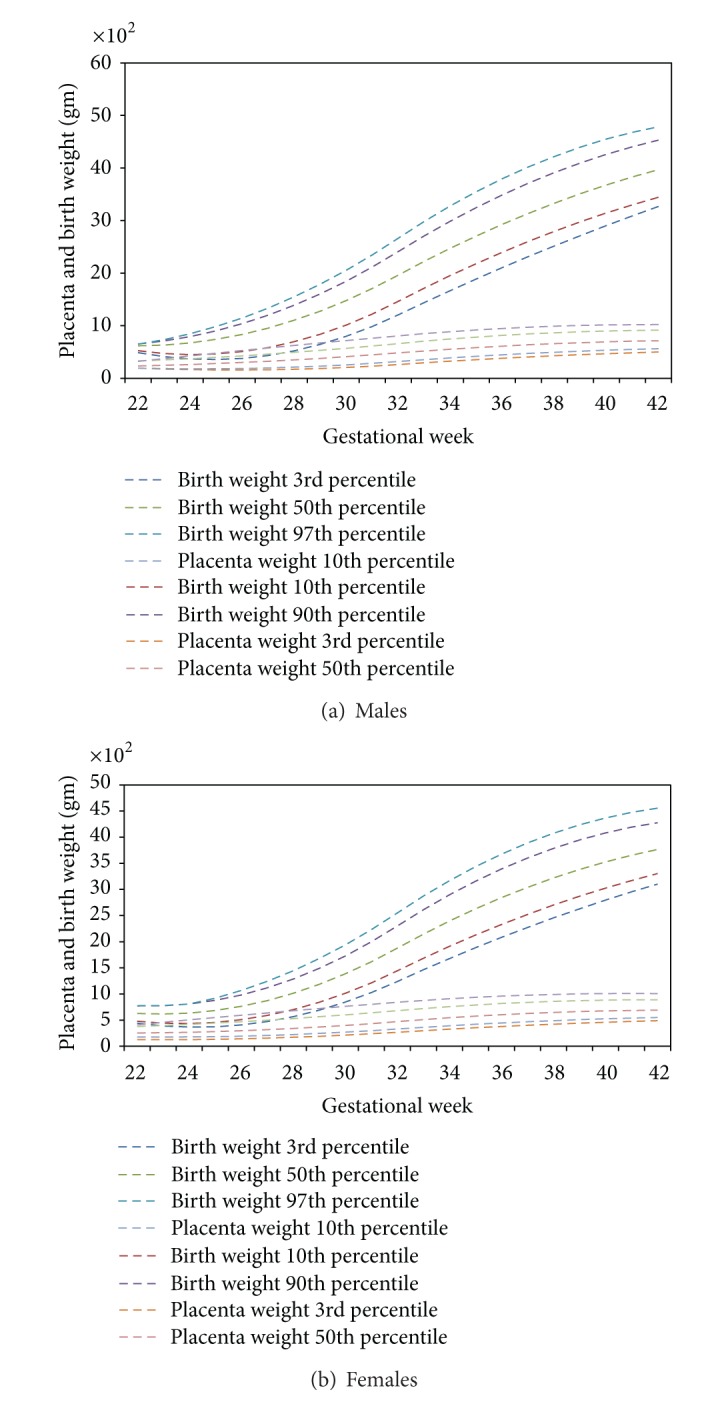
Inclusive of regional referrals placenta and birth weight percentile distributions by gestational age.

Figures 2(a) and 2(b) present placental weight and birth weight distributions for males and females, respectively. Because these curves are for the last half of gestation, placental growth has to some degree leveled off while fetal growth continues at an accelerated pace. Changes in birth weight and placental weight across the study period were examined and we found no differences (data not shown).

**Figure 3:**
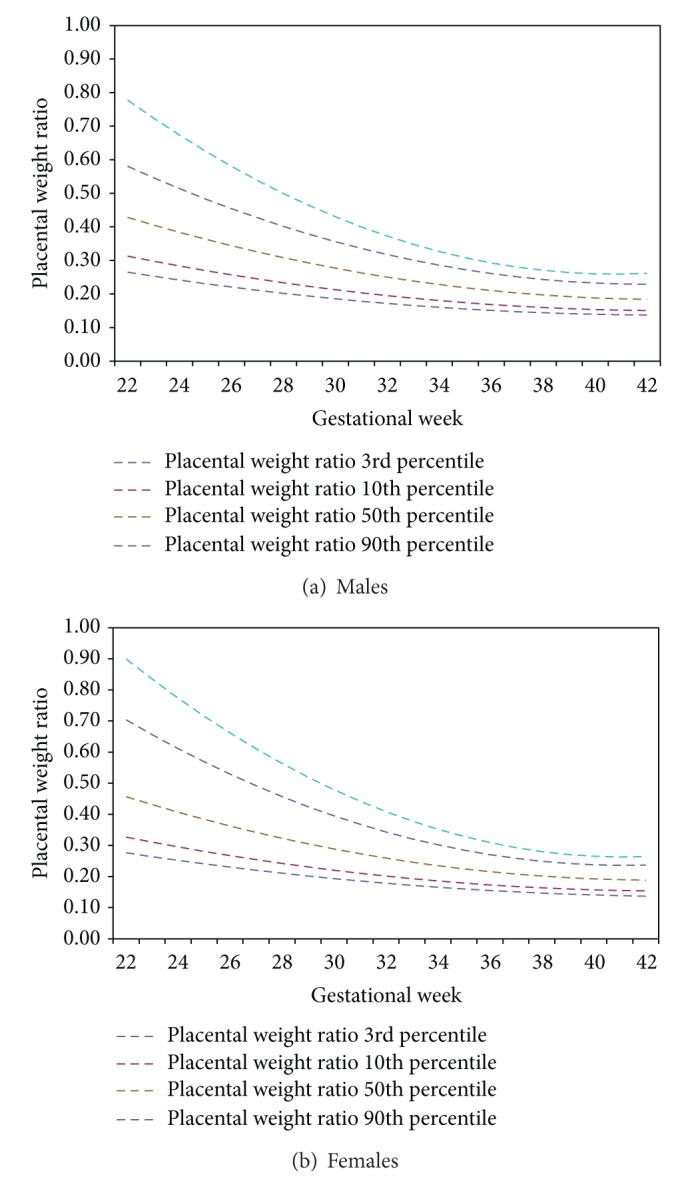
Inclusive of regional referrals PWR distributions by gestational age.

Additional file 1.xlsx and Additional file 2.xlsx in Supplementary Material available online at http://dx.doi.org/10.1155/2014/291846 present the PWR standards when inclusion criteria are relaxed to include regional referrals in the sample. All of the percentiles achieved a good fit across the range of gestational ages and reached a statistical significance of *P* < 0.001. Comparing the citywide population to the total sample revealed them to be similar, with minute differences presenting themselves at the extreme percentiles at the earlier gestational ages. Furthermore, comparing the 10th and 90th percentiles, which are often used as cut-off points, revealed almost no differences, even at the earlier gestational ages. The distributions of the PWR curves for males and females are illustrated in Figures 3(a) and 3(b), for the total population including regional referrals. The PWR decreases as gestational increases. In general, the females have higher PWRs than males. The slightly higher PWR in females than in males is consistent across percentiles. The ranges for the PWR are greatest at the highest percentiles. For both males and females, the ranges at the 90th percentile are more than 2 times as wide as at the 10th percentile.

### 3.2. Placental Weight Ratio Distribution Curves Stratified by SGA, AGA, and LGA Status

**Figure 4:**
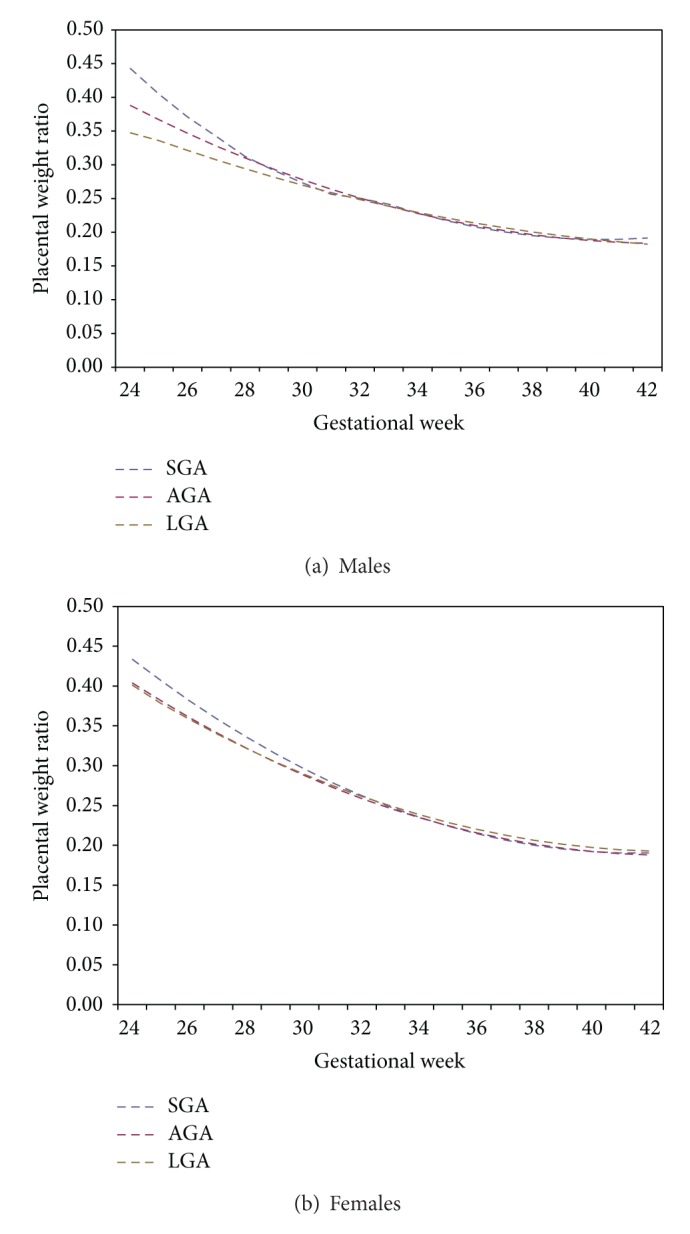
Inclusive of regional referrals SGA, AGA, and LGA median PWR distributions by gestational age.

PWR distributions for the entire sample, inclusive of regional referrals, were used in an analysis of SGA, AGA, and LGA. The proportion with PWRs <10th percentile between the 10th and the 90th percentile and >90th percentile are presented in Table 1. There are a higher proportion of SGA infants for both males and females in the extreme PWR groups. Furthermore, there are fewer LGA infants in the lowest PWR group. The median PWR curves for each of SGA, AGA, and LGA are presented in Figures 4(a) and 4(b). At the earlier gestational ages both male and female SGA infants have higher PWRs than male and female AGA and LGA infants. However, the PWRs at term gestations are nearly identical in both SGA and LGA infants. In fact, LGA infants have slightly higher median ratios at term than both SGA and AGA infants.

## 4. Discussion

The results of this study contribute to the current literature by creating gender-specific PWR percentile standards. While PWR is an important indicator of fetal health, there are few population standards for comparison. A few previous studies have presented the relation between placental weight and birth weight and only two of these reported percentiles curves for the PWR [6, 20]. Thompson et al. [20] reported placental percentile curves for a Norwegian population, and Almog et al. [6] presented PWR curves for a Canadian population. Comparison of our results with Almog's Canadian standards reveals close resemblance between the two populations, such as median 40-week PWRs (0.1938 and 0.19 for males and 0.1981 and 0.20 for females, resp.). Dombrowski et al. [21] published data on placental weight and PWR in North American population. However, their study is based on data from 1984 to 1991, over two decades ago and contained data mostly on a black population (81.4%), so the results cannot reasonably be compared. Compared to the only other available set of PWR percentiles in a Canadian population, [6] our results complement this literature and now provide more precise PWR predictions, particularly at the extreme percentiles, due to our larger sample size.

Our standards include earlier gestational ages than both of the aforementioned studies. Both of the abovementioned studies have gestational age standards starting at 24 weeks; however, our standards provide estimates starting at 22 weeks. Comparison of our results to Thompson et al.'s [20] shows that our <10th percentile estimates are lower than theirs, but our >90th percentile estimates are higher than their standards. However, comparisons of our results inclusive of regional referrals to Almog's curves reveal very similar standards. Our results have slightly lower PWRs at all gestational ages and percentiles [6].

A declining PWR with increasing gestational age reported here is similar to that described by others [21–23]. The placenta and fetus follow different growth patterns during gestation [3]. The placenta follows an S-shaped growth curve whereas fetal growth follows an exponential pattern in mid pregnancy, with most growth occurring in a linear fashion during the third trimester [3]. In the earlier gestational ages the birth weight is low in comparison to the placental weight as a result of the higher growth rate of the placenta earlier in gestation. Moreover, our placental growth curves show how the majority of placental growth occurs before 33 weeks of gestation. This accounts, at least in part, for the higher PWRs at earlier gestations. Previous authors have shown that the placenta responds to the interruption of the fetal villous circulation in the first half of gestation by initiating compensatory hyperplasia [24]. The majority of placental growth occurs at the earlier gestational ages; therefore, this is where the greatest differentiation of PWRs is expected.

Our birth weight distributions differ from the Kramer et al. [18] birth weight distributions in that our birth weights are somewhat larger. This might be expected since our population included more recent data and birth weight is increasing over time due to increases in maternal anthropometry, reduced cigarette smoking, and changes in sociodemographic factors [25]. Also, Kramer's curves did not include the Ontario population [18]; therefore, the reference populations are somewhat different.

Our PWR curves are similar whether inclusive or exclusive of the referral population. This may be because, at earlier gestations, the vast majority of regional births occur in this tertiary referral center. Thus, the lower gestations represent a “whole population.” At later gestational ages, where one might expect the referral population to represent a biased sample of higher risk births, the actual numbers contributed by regional referrals are much smaller and would not substantially affect the percentile estimates for term and near-term births. Thus, the results are not unduly biased by inclusion of regional births. This is important because their inclusion had a sample size advantage, as it allowed us to use the total population for analyses stratified by fetal growth adequacy.

### 4.1. Stratification by Fetal Growth Adequacy

An additional novelty is the examination of percentile curves stratified by fetal growth adequacy, specifically focusing upon how PWRs change across gestational age between SGA, AGA, and LGA infants.

The literature suggests that a higher proportion of LGA infants have placenta weights above the 90th percentile and a lower share of placental weights below the 10th percentile than SGA and AGA infants [26]. Furthermore, PWRs have been found to be the lower in LGA infants than in AGA and SGA infants [27]. Our results show that, at earlier gestational ages in male infants, LGA infants generally have lower PWRs than AGA infants. This pattern holds true across all percentiles until the 33rd week of gestation, when the LGA and AGA standards become more similar. However, the differences between the LGA and AGA standards are not as pronounced as the differences between the SGA and AGA standards.

Previous studies have indicated that overall SGA infants have higher PWRs [3, 5] and that SGA infants have a higher proportion of placental weights at both extremes, but none of these studies have looked at the relationships across gestation or between percentiles [4, 16, 26, 28, 29].

Our curves show that as gestational age advances, the PWRs become more similar between SGA, AGA, and LGA infants, yet the PWR is still higher in female SGA infants. At earlier gestational ages the SGA standards are much higher than the AGA standards. This suggests that SGA may be detectable early in pregnancy. Therefore, the SGA infant is generally under grown in relation to placental size, suggesting placental function, rather than size, constraints for fetal growth. Salafia et al. [1] showed that an elevated PWR might be an indication of an inefficient placenta with a reduced ability to maintain fetal growth. Indeed Kingdom and Kaufmann [30] report that preplacental or uteroplacental hypoxia with adaptive placental growth is a primary cause for growth restriction at term. However, the nonplacental chorion and amnion also contribute to overall placental weight, and more so for SGA infants; [23] this may also account, at least in part, for the higher PWR of infants in the SGA group. On the other hand, low PWRs are indicative of an increased efficiency of the placentas of the smaller fetuses, whereas, high PWRs are indicative of a potential failed compensation [17, 31–36]. The adaptive response in the placenta enhances transfer of substrates from the mother to fetus and improves efficiency of substrate utilization. The placenta compensates to minimize fetal growth restriction. However, placental function is not always improved with increases in weight. This may be due in part to the increase in syncytial knots especially in hypertensive pregnancies, which is an attempt at placental hyperplasia. The difference being that there is no associated functional angiogenesis, and therefore, no functional placental tissue in terms of oxygen and nutrient transfer [1, 37–41]. Therefore, it is suggested that the PWR can be used as a predictor for placental functional efficiency. Based on these suggestions, and the fact that our results show that SGA infants have a higher PWR than AGA and LGA infants, we propose that this may be due to a failed compensation of the placenta in SGA infants, whereas the lower PWRs seen in the SGA infants are indicative of either a biological tendency to smaller stature or an adaptive increase in placental efficiency.

A major strength of the study is the available sample size. The perinatal database provided a large number of observations with matching placental weight, birth weight, and gestational age. This allowed for the creation of accurate standards, and for the resulting percentile curves to be stratified by fetal growth adequacy standard.

Birth weights vary widely from country to country [18, 42] and as such it might be considered appropriate that birth weight percentiles should be based on data from the actual country or at least from a comparable country. This is often not the case and can lead to inappropriate use of the percentiles in a population where the distribution of birth weight is shifted, particularly to the left. Therefore, our results may be considered generalizable to other urban centers in Western based cultures. Also, the study of placental weight at the time of delivery is a crude measure of placental growth and development. However, when it is collected in a routine manner and related to birth weight, it provides information of biological importance.

## 5. Conclusions

The PWR distribution curves provide a standard that researchers can apply as a reference to identify infants who have abnormal PWRs. Identifying infants with high PWRs is important for patient care in both the short and long term. These analyses have included birth weight, placental weight, and even the PWR; however, the relative magnitude of the latter, in terms of percentiles, has not been previously available for all gestational ages in a Canadian population.

## Supplementary Material

The supplementary material presents the placental weight ratios, inclusive of regional referrals, for both males and females at each percentile. They also include the sample sizes at each gestational age.

## Figures and Tables

**Table 1:** Placenta weight ratio distributions for SGA, AGA, and LGA infants based upon the inclusive of regional referrals standards.

Inclusive of regional referrals standards	Expected %	Males			Females		
		SGA	AGA	LGA	SGA	AGA	LGA
>90th	10%	13.18%	9.74%	9.82%	11.68%	9.66%	11.69%
10–90th	80%	74.42%	80.22%	81.64%	77.56%	80.22%	80.14%
<10th	10%	12.10%	10.04%	8.53%	10.76%	10.12%	8.16%

## Abbreviations

PWR: Placental weight ratio
SGA: Small for gestational age
AGA: Average for gestational age
LGA: Large for gestational age.
