# Supplementary material for: Population-Based Placental Weight Ratio Distributions
Source: Int J Pediatr. 2014 May 6;2014:291846. doi: 10.1155/2014/291846 (PMC4033358; doi:10.1155/2014/291846)
Supplement: Supplementary file 1 — The supplementary material presents the placental weight ratios, inclusive of regional referrals, for both males and females at each percentile. They also include the sample sizes at each gestational age. [file 291846.f1.docx]

**Table 1:** Inclusive of Regional Referrals Male Placental Weight Ratio Curves by Gestational Age for the 3^rd^ through the 97^th^ Percentile

| **Gestational Age** | **n (%)** | **3rd Percentile** | **5th Percentile** | **10th Percentile** | **25th Percentile** | **50th Percentile** | **75th Percentile** | **90th Percentile** | **95th Percentile** | **97th Percentile** |
| --- | --- | --- | --- | --- | --- | --- | --- | --- | --- | --- |
| **22** | 11 (0.05) | 0.2646 | 0.2925 | 0.3127 | 0.3685 | 0.4281 | 0.5069 | 0.5803 | 0.6849 | 0.7777 |
| **23** | 17 (0.08) | 0.2526 | 0.2783 | 0.2976 | 0.3495 | 0.4052 | 0.4780 | 0.5461 | 0.6405 | 0.7239 |
| **24** | 26 (0.12) | 0.2413 | 0.2648 | 0.2832 | 0.3314 | 0.3835 | 0.4505 | 0.5136 | 0.5985 | 0.6731 |
| **25** | 44 (0.21) | 0.2305 | 0.2519 | 0.2696 | 0.3142 | 0.3629 | 0.4244 | 0.4829 | 0.5589 | 0.6252 |
| **26** | 30 (0.14) | 0.2204 | 0.2398 | 0.2568 | 0.2980 | 0.3435 | 0.3998 | 0.4540 | 0.5216 | 0.5802 |
| **27** | 40 (0.19) | 0.2108 | 0.2285 | 0.2446 | 0.2827 | 0.3251 | 0.3767 | 0.4268 | 0.4867 | 0.5382 |
| **28** | 53 (0.25) | 0.2018 | 0.2178 | 0.2332 | 0.2684 | 0.3079 | 0.3551 | 0.4014 | 0.4541 | 0.4991 |
| **29** | 57 (0.29) | 0.1934 | 0.2078 | 0.2225 | 0.2549 | 0.2917 | 0.3349 | 0.3777 | 0.4240 | 0.4630 |
| **30** | 70 (0.33) | 0.1855 | 0.1985 | 0.2126 | 0.2425 | 0.2767 | 0.3162 | 0.3557 | 0.3961 | 0.4298 |
| **31** | 96 (0.45) | 0.1783 | 0.1900 | 0.2034 | 0.2309 | 0.2628 | 0.2989 | 0.3355 | 0.3707 | 0.3996 |
| **32** | 103 (0.49) | 0.1716 | 0.1821 | 0.1949 | 0.2203 | 0.2501 | 0.2832 | 0.3171 | 0.3476 | 0.3723 |
| **33** | 165 (0.78) | 0.1655 | 0.1750 | 0.1872 | 0.2106 | 0.2384 | 0.2688 | 0.3004 | 0.3269 | 0.3479 |
| **34** | 178 (0.84) | 0.1600 | 0.1686 | 0.1802 | 0.2019 | 0.2279 | 0.2560 | 0.2854 | 0.3086 | 0.3265 |
| **35** | 323 (1.52) | 0.1551 | 0.1628 | 0.1739 | 0.1941 | 0.2184 | 0.2446 | 0.2722 | 0.2926 | 0.3081 |
| **36** | 643 (3.03) | 0.1507 | 0.1578 | 0.1683 | 0.1872 | 0.2101 | 0.2347 | 0.2608 | 0.2790 | 0.2925 |
| **37** | 1587 (7.47) | 0.1470 | 0.1535 | 0.1635 | 0.1813 | 0.2030 | 0.2262 | 0.2511 | 0.2678 | 0.2800 |
| **38** | 3848 (18.10) | 0.1438 | 0.1499 | 0.1594 | 0.1763 | 0.1969 | 0.2192 | 0.2431 | 0.2590 | 0.2703 |
| **39** | 5597 (26.33) | 0.1412 | 0.1470 | 0.1561 | 0.1722 | 0.1919 | 0.2137 | 0.2369 | 0.2525 | 0.2637 |
| **40** | 5344 (25.14) | 0.1392 | 0.1448 | 0.1535 | 0.1691 | 0.1881 | 0.2096 | 0.2325 | 0.2483 | 0.2599 |
| **41** | 2974 (13.99) | 0.1378 | 0.1434 | 0.1516 | 0.1669 | 0.1854 | 0.2070 | 0.2298 | 0.2466 | 0.2591 |
| **42** | 49 (0.23) | 0.1370 | 0.1426 | 0.1504 | 0.1657 | 0.1838 | 0.2059 | 0.2288 | 0.2472 | 0.2613 |

**Table 2:** Inclusive of Regional Referrals Female Placental Weight Ratio Curves by Gestational Age for the 3^rd^ through the 97^th^ Percentile

| **Gestational Age** | **n (%)** | **3rd Percentile** | **5th Percentile** | **10th Percentile** | **25th Percentile** | **50th Percentile** | **75th Percentile** | **90th Percentile** | **95th Percentile** | **97th Percentile** |
| --- | --- | --- | --- | --- | --- | --- | --- | --- | --- | --- |
| **22** | 7 (0.03) | 0.2763 | 0.2886 | 0.3266 | 0.3777 | 0.4566 | 0.5596 | 0.7037 | 0.8268 | 0.8995 |
| **23** | 11 (0.05 | 0.2639 | 0.2759 | 0.3106 | 0.3585 | 0.4312 | 0.5252 | 0.6563 | 0.7691 | 0.8348 |
| **24** | 24 (0.12) | 0.2522 | 0.2637 | 0.2955 | 0.3402 | 0.4070 | 0.4926 | 0.6114 | 0.7144 | 0.7736 |
| **25** | 28 (0.14) | 0.2410 | 0.2522 | 0.2810 | 0.3229 | 0.3841 | 0.4618 | 0.5690 | 0.6628 | 0.7159 |
| **26** | 34 (0.17) | 0.2304 | 0.2412 | 0.2674 | 0.3064 | 0.3625 | 0.4327 | 0.5292 | 0.6142 | 0.6616 |
| **27** | 36 (0.18) | 0.2203 | 0.2308 | 0.2545 | 0.2909 | 0.3421 | 0.4055 | 0.4919 | 0.5687 | 0.6108 |
| **28** | 48 (0.24) | 0.2108 | 0.2209 | 0.2424 | 0.2764 | 0.3230 | 0.3801 | 0.4572 | 0.5262 | 0.5635 |
| **29** | 55 (0.27) | 0.2019 | 0.2117 | 0.2311 | 0.2627 | 0.3052 | 0.3564 | 0.4250 | 0.4867 | 0.5196 |
| **30** | 56 (0.28) | 0.1935 | 0.2030 | 0.2205 | 0.2499 | 0.2886 | 0.3346 | 0.3953 | 0.4503 | 0.4792 |
| **31** | 68 (0.34) | 0.1857 | 0.1949 | 0.2107 | 0.2381 | 0.2733 | 0.3145 | 0.3682 | 0.4170 | 0.4422 |
| **32** | 73 (0.36) | 0.1785 | 0.1873 | 0.2017 | 0.2272 | 0.2593 | 0.2963 | 0.3436 | 0.3866 | 0.4087 |
| **33** | 104 (0.52) | 0.1718 | 0.1804 | 0.1934 | 0.2172 | 0.2465 | 0.2798 | 0.3215 | 0.3594 | 0.3787 |
| **34** | 148 (0.73) | 0.1657 | 0.1740 | 0.1859 | 0.2082 | 0.2350 | 0.2651 | 0.3020 | 0.3351 | 0.3521 |
| **35** | 242 (1.20) | 0.1602 | 0.1681 | 0.1792 | 0.2000 | 0.2248 | 0.2523 | 0.2850 | 0.3139 | 0.3290 |
| **36** | 516 (2.56) | 0.1552 | 0.1629 | 0.1733 | 0.1928 | 0.2158 | 0.2412 | 0.2705 | 0.2958 | 0.3093 |
| **37** | 1390 (6.89) | 0.1508 | 0.1582 | 0.1681 | 0.1865 | 0.2081 | 0.2319 | 0.2586 | 0.2806 | 0.2931 |
| **38** | 3652 (18.10) | 0.1469 | 0.1541 | 0.1637 | 0.1811 | 0.2017 | 0.2244 | 0.2492 | 0.2686 | 0.2804 |
| **39** | 5393 (26.72) | 0.1436 | 0.1506 | 0.1601 | 0.1766 | 0.1965 | 0.2187 | 0.2424 | 0.2595 | 0.2712 |
| **40** | 5407 (26.79) | 0.1409 | 0.1477 | 0.1572 | 0.1731 | 0.1926 | 0.2148 | 0.2380 | 0.2535 | 0.2653 |
| **41** | 2857 (14.15) | 0.1388 | 0.1453 | 0.1552 | 0.1704 | 0.1900 | 0.2127 | 0.2363 | 0.2506 | 0.2630 |
| **42** | 37 (0.18) | 0.1372 | 0.1435 | 0.1538 | 0.1687 | 0.1886 | 0.2124 | 0.2370 | 0.2507 | 0.2641 |
